# Treatment of acromegaly patients at the Federal University of Triângulo Mineiro (UFTM): Experience Report

**DOI:** 10.6061/clinics/2017(04)05

**Published:** 2017-04

**Authors:** Maria de Fátima Borges, Beatriz Hallal Jorge Lara, Janaíne Machado Tomé, Leopoldo Prezia de Araújo, Flávio Cesar Lucas Bugiga, Júlio Cláudio Sousa, Jacqueline Mendes Fonseca Soares, Roberto Alexandre Dezena, Beatriz Pires Ferreira

**Affiliations:** I Divisão de Endocrinologia e Metabolismo, Universidade Federal do Triângulo Mineiro, Uberaba, MG, BR; II Divisão de Neurocirurgia, Universidade Federal do Triângulo Mineiro, Uberaba, MG, BR; III Divisão de Otorrinolaringologia, Universidade Federal do Triângulo Mineiro, Uberaba, MG, BR

**Keywords:** Acromegaly, Gigantism, Treatment, Outcome

## Abstract

**OBJECTIVE:**

To evaluate the effectiveness of the treatment of acromegaly patients at the Federal University of Triangulo Mineiro.

**METHODS:**

Cross-sectional and retrospective study of thirty cases treated over a period of two decades.

**RESULTS:**

17 men (56.7%) aged 14-67 years and 13 women aged 14-86 years were analyzed. Twenty-one patients underwent transphenoidal surgery, whichwas associated with somatostatin receptor ligands in 11 patients (39.3%), somatostatin receptor ligands + radiotherapyin 5 patients (17.8%), radiotherapy in 3 patients (10.7%), and radiotherapy + somatostatin receptorligands + cabergoline in 1 patient (3.6%). Additionally, 2 patients underwent radiotherapy and surgeryalone. Six patients received somatostatin receptor ligands before surgery, and 2 were not treated due to refusal and death. Nine patients have died, and 20 are being followed; 13 (65%) have growth hormonelevels o1 ng/mL, and 11 have normal insulin-like growth factor 1 levels.

**CONCLUSION:**

The current treatment options enable patients seen in regional reference centers to achieve strict control parameters, which allows them to be treated close to their homes.

## INTRODUCTION

Classically described as a rare disease 1, acromegaly has an estimated prevalence of 36-60 cases/million persons and an annual incidence of 3-4 cases/million persons, and its incidence triples among adults aged 65 years and older 2,3. Men and women are similarly affected, and the most common cause is a growth hormone (GH)-secreting pituitary macroadenoma 1-3.

The diagnosis of acromegaly is based on findings such as panhypopituitarism, neuro-ophthalmic symptoms and signs due to compression of adjacent structures as the tumor grows and confirmed by increased GH and insulin-like growth factor 1 (IGF-1) levels, non-suppression of GH during an oral glucose tolerance test (OGTT) and magnetic resonance imaging of the sella turcica 1,2,4.

Treatment of acromegaly is considered effective when patients achieve basal plasma GH levels below 1 ng/mL or glucose-suppressed GH levels below 0.4 ng/mL and normal IGF-1 levels 5-7. Currently, these hormonal control targets can only be achieved by following a treatment plan consisting of tumor ablation either preceded or followed by pharmacological treatment with increasing individualized doses of somatostatin receptor ligands (SRLs) and, in some cases, dopamine receptor agonists and GH receptor antagonists 4,8-10. Radiotherapy is a third-line treatment and should be performed when surgery and drug therapies are not effective or when aggressive tumors invade the surrounding structures 4,6-10.

The treatment of acromegaly has evolved considerably over the past three decades 11, allowing patients to be treated outside the major referral centers in Brazil. The objective of this study was to report our experience with patients with acromegaly or gigantism who were treated at the Federal University of Triângulo Mineiro (Universidade Federal do Triângulo Mineiro – UFTM) and to evaluate the effectiveness of treatment and the outcomes of these patients. UFTM is located in Uberaba, a medium-sized city in the state of Minas Gerais, Brazil. The patients outcomes were evaluated according to the hormonal and metabolic control criteria currently established in the literature 4-7. The most common co-morbidities and complications were also analyzed.

## SUBJECTS AND METHODS

This retrospective study was approved by the Research Ethics Committee of UFTM and was conducted by the Departments of Neurosurgery and Endocrinology.

The medical records of patients with acromegaly and gigantism who were treated in these departments were analyzed. The medical records were actively searched, and a summary was completed for each case. The summary included the patient's clinical history with signs and symptoms reported by the patient, the results of a standardized diagnostic investigation performed at the Department of Endocrinology, a description of treatments performed and their complications, a description of the evolution of GH and IGF-1 levels during treatment and of the control of adenohypophyseal hormone levels at diagnosis and during replacement therapy, the presence, treatment and control of metabolic complications such as diabetes, hypertension and dyslipidemia and references to cardiorespiratory, joint, and gastrointestinal complications, thyroid diseases and cancer. The medical records of deceased patients and the causes of their deaths were also analyzed.

The effectiveness of treatment was analyzed according to the strict control criteria recommended by the consensus described in the literature: basal baseline serum concentrations of GH <1 ng/mL or GH <0.4 ng/mL during to a glucose tolerance test and IGF-1 < two standard deviations (SD) from the mean for age and gender 6,7.

Over a 23-year period, 27 patients with acromegaly and 3 with gigantism were diagnosed. These individuals were divided into group 1 (G1), which consisted of 17 men (56.7%) and group 2 (G2), which consisted of 13 women (43.3%). The overall male-female ratio of the patients in the study was 1.3:1; their ages at diagnosis ranged from 14 to 67 years (median 45.5 years) and from 14 to 86 years (median 47.5 years) for males and females, respectively.

### Statistical analysis

Anthropometric data and hormone concentrations are presented as median, minimum and maximum values. Student's *t*-test was used to compare the age and body mass index (BMI) of females and males at diagnosis. Friedman's test followed by Dunn's multiple comparison test was used to compare the patients' GH and IGF-1 levels during the treatment stages. The Mann-Whitney U test was used to compare the GH levels measured in the last clinical evaluation between patients who underwent surgery + radiotherapy + SRLs and those who underwent surgery + SRLs. Spearman's test was used for correlations between GH levels before treatment and GH levels after surgery or after radiotherapy, and between GH basal values and current GH values of patients treated with surgery + radiotherapy + SRLs and among patients who received surgery +SRLs. *P*-values less than 0.05 were considered significant.

## RESULTS

Of the 30 patients, 27 had excessive GH production due to macroadenomas, and 3 patients had excessive GH production caused by microadenomas. After diagnosis, 1 patient refused surgical or medical treatment. For the remaining 29 patients, the follow-up period ranged from 4 months (newly diagnosed patients) to 23 years (mean 9 years). During this period, 9 patients died, and 20 remained on routine follow-up.

The BMI of the G1 and G2 groups ranged from 17.6 to 53.4 kg/m^2^ (median 28.4) and from 25.5 to 29.8 kg/m^2^ (median 27.8), respectively. Of the total, 25 patients had BMIs indicating overweight (n=18) or obesity (n=7). No significant differences were observed between men and women regarding age at diagnosis (*p*=0.973) or BMI (*p*=0.425) (Table 1).

The majority of the patients were from Uberaba (n=15) or municipalities of the Triângulo Sul region (n=12). These regions are covered by the Clinics Hospital/UFTM for highly complex procedures through the Unified Health System (Sistema Único de Saúde - SUS) 12. Three patients were from São Paulo state and lived in towns bordering the state of Minas Gerais.

The mean time to diagnosis was 4 years and ranged from at the first doctor visit to 20 years. Most patients reported symptoms/signs classically associated with acromegaly (Table 2).

Initial laboratory tests indicated GH levels ranging from 8.0 to 355.0 ng/mL (median 25.6 ng/mL) and IGF-1 levels (analyzed according to age group) between 105.0 and 1321.0 ng/mL (median 707.5 ng/mL). Four patients (1 man and 3 women) had mixed GH- and prolactin-secreting tumors.

The therapeutic approach is shown in Table 3. The treatment approaches were individualized to achieve the control criteria. One male patient who refused treatment and 1 female patient diagnosed at age 86 who died in the first months of follow-up are not included in the treatment table. Transphenoidal adenomectomy was the first approach for most patients (21/28, 75%). Complementary radiotherapy was offered to patients up to the 1990s, and 9 patients received it. One patient underwent radiotherapy alone and another patient underwent surgery alone and achieved clinical control.

When SRL began to be provided by the SUS, its administration became the main approach used to complement surgery. SRL was initially administered subcutaneously (100 µg every 8 hours), and beginning in the 1990s, all patients received octreotide LAR (Novartis®). Over the past year, 3 patients used lanreotide (Ipsen®) due to poor adherence to medication.

Of the 28 patients, 17 (60.7%) received clinical treatment after surgery, and 6 patients (21.4%) received clinical treatment prior to surgery. Four patients who presented elevated prolactin levels were also treated with cabergoline (Pfizer®).

Of the 21 patients who underwent surgery, only 1 patient did not require therapeutic complementation to achieve the clinical control criteria. For the other 20 patients, the reduction in the absolute and percent GH levels compared to the baseline value at diagnosis was analyzed. This analysis could not be performed for IGF-1 levels due to missing baseline values (Table 4).

Surgery resulted in a 12.7-83.0% (median 53.0%) reduction in basal GH levels, but there was no significant difference between GH levels before and after surgery (*p*>0.05). GH levels were reduced by 14.0-96.8% (median 87.7%) among patients who underwent radiotherapy after surgery (n=9), but no significant difference was observed between GH levels at baseline and post-surgery (*p*>0.05). Six of these patients were treated with SRLs, and a significant reduction in GH levels was observed (*p*<0.05), with percent changes ranging from 90.4 to 99.5% (median 98.7%) (Table 4).

In 11 cases, surgical treatment was complemented only with SRLs. In these patients, GH levels were reduced by 65.0-99.9% (median 95.2%) compared to baseline levels, and the reduction was significant (*p*<0.05). When the reduction in GH level was compared between therapeutic modalities, no differences were observed at the end of treatment between surgery + radiotherapy + SRLs and surgery + SRLs (*p*>0.05). However, the reductions achieved with surgery + SRLs were significant compared to surgery + radiotherapy (Table 4).

**Figure 1:**
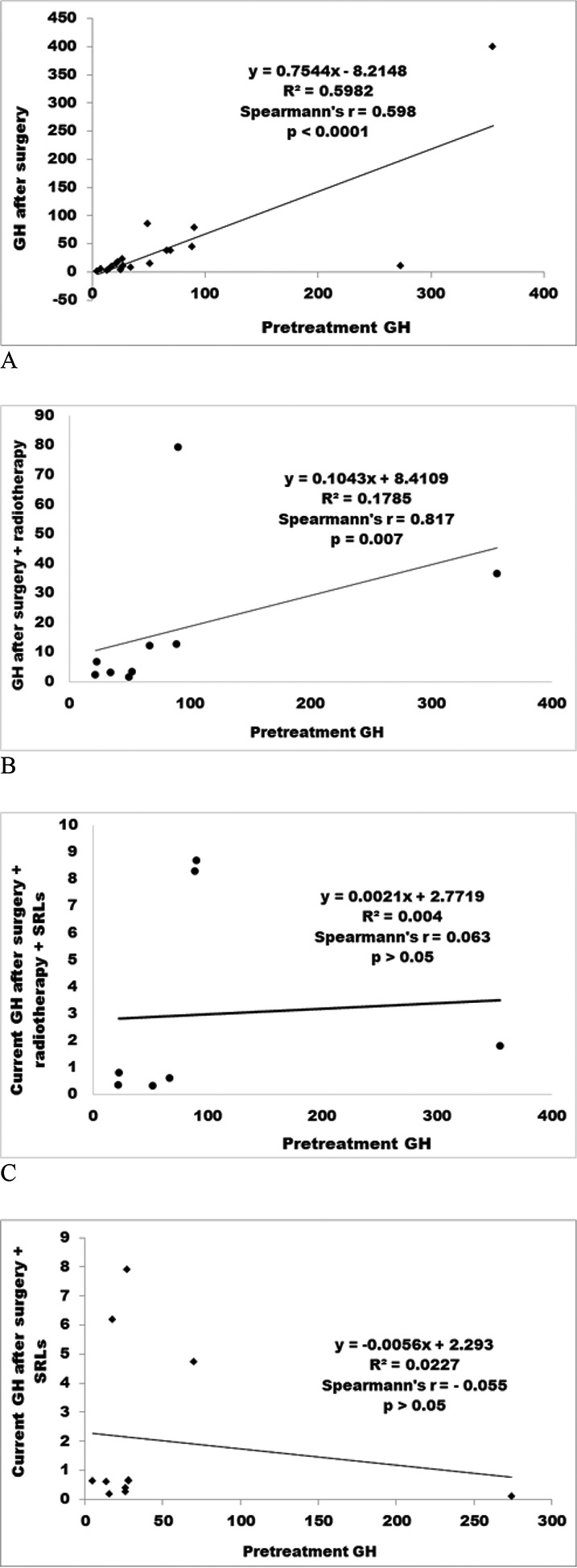
Correlations between pretreatment GH levels and GH levels obtained after different treatment modalities: after surgery (A); after surgery + radiotherapy (B); after surgery + radiotherapy + SRLs (C); and after surgery + SRLs (D).

No significant correlation was found between the pretreatment and current GH and IGF-1 levels among patients who received surgery + radiotherapy + SRLs, or among those who received surgery + SRLs. A positive and significant correlation was observed between pretreatment GH levels and postsurgical GH levels, as well as between pretreatment GH levels and GH levels after surgery + radiotherapy (Figure 1).

Nine deaths occurred during the follow-up period, and 20 patients remain on routine follow-up. The treatment modalities of the remaining patients are shown in Table 5; surgery followed by clinical treatment was the main treatment approach and clinical treatment was also provided while the patient awaited surgery.

A recent evaluation revealed that the strict control criteria were met in 11/20 of the remaining patients (55.0%) (Table 6). In 2 cases, baseline GH levels were less than 1.0 ng/mL, with IGF-1 >2 SDs above the mean for age and gender. Two female patients with GH >1.0 and <2.5 ng/mL had levels near the control target. Among the patients with GH >2.5 and elevated IGF-1, 3 male patients had postoperative recurrence, and 2 were recently diagnosed and are being primarily treated with SRLs.

Among the deceased patients (n=9), the age at death was 44-87 years (median 54 years), and the follow-up period was 1-23 years (median 6 years). Of these patients, only 2 had GH/IGF1 levels that met the strict control criteria. Six patients died due to infections (bronchopneumonia and urinary tract infections), 2 died due to cardiovascular events (acute myocardial infarction and pulmonary embolism), and 1 patient died due to breast cancer.

### Co-morbidities and complications

In addition to the overweight and obesity mentioned previously, 30 patients had other co-morbidities: 11 patients (36.7%) presented impaired fasting glucose, 7 patients (23.3%) had diabetes, and 7 patients developed diabetes during follow-up. Seventeen patients (56.7%) showed reduced HDL-cholesterol levels, 11 patients (36.7%) showed increased LDL-cholesterol levels, and 8 patients (26.7%) showed increased triglyceride levels.

Postoperative complications were rarely described. One patient had a cerebrospinal fluid leak, and 2 had transient diabetes insipidus. Regarding clinical treatment, the most common complaint was local pain and discomfort on the day of administration of the medication. Three patients tended to non-adherence to drug treatment; thus, octreotide was replaced by lanreotide in the past year. Two patients complained of abdominal pain and increased bowel movements.

The most common chronic complications observed among the patients were analyzed. The thyroid was evaluated in 26 patients. Two cases of papillary cancer, 2 cases of follicular adenomas and 2 cases of chronic autoimmune thyroiditis were found. Colonoscopies were performed in 12 patients, and 4 cases of colon polyps and 2 cases of diverticular disease were found.

The cardiovascular assessments revealed that 12/30 patients had hypertension, which was controlled with losartan, amlodipine and atenolol. Among these patients, left ventricular overload was diagnosed on ECG, and left ventricular hypertrophy was diagnosed on conventional echocardiography.

Joint abnormalities, such as arthritis of the large joints and spine, were observed in 25 patients. Two patients had carpal tunnel syndrome, and 1 patient with gigantism had epiphysiolysis of the femoral head.

## DISCUSSION

This study is unique because it reports the diagnosis, treatment and follow-up of patients with acromegaly/gigantism outside of major referral medical centers in Brazil. Most of our patients were from Uberaba and nearby municipalities, and the resources available at the Clinics Hospital/UFTM allowed them to be treated close to their homes, which enabled regular monitoring and follow-up.

The incidence and prevalence of acromegaly in this region are unknown. The analyzed cases span 23 years; objectively, 2-3 cases have been identified per year over the past 10 years, indicating our service as a regional referral center that covers a population of 700,000 inhabitants 13. These numbers show that this region needs educational campaigns for early recognition of the disease and its diagnosis.

Treatment of acromegaly/gigantism has advenced greatly in recent decades, and studies indicate that the strict control of GH/IGF-1 levels reduces morbidity and mortality 5,10,11. The subjects in the present study reflect this evolution. The first patient was treated with radiotherapy alone. Until the 1990s, transphenoidal surgery was systematically followed by radiotherapy. However, since the distribution of octreotide by the SUS, radiotherapy in generally used only in exceptional cases, as described by Jallad et al. 14.

Most of the acromegaly patients analyzed in the present study were diagnosed late despite the fact that they displayed the classic symptoms and signs of the disease. In most cases, the etiology was a pituitary macroadenoma, which was on average diagnosed in the fifth decade of life. These findings demonstrates the insidious nature of the disease, which often leads to late diagnosis at an advanced stage. In most cases, hyperproduction of GH began after epiphyseal bone closure, as shown by the fact that the acromegaly/gigantism ratio was 9:1. Acromegaly occurred in both men and women, with a slight predominance in men (1.3:1), consistent with reposts in the literature 15-17.

Most of our patients were primarily treated with surgery (21/30). Current guidelines suggest that pituitary surgery should be the primary approach to the disease due to its low complication rate and to achieve debulking of the pituitary tumor 6,7. Although Buchfelder & Schlaffer^18^ reported a 74% success rate in achieving the GH/IGF-1 level control parameters, the stricter current parameters 6,7 led Evran et al. 19 and Sarkar et al. 20 to report success rates of 20-50% depending on the size of the tumor, the presence and degree of cavernous sinus invasion, the surgical technique employed and the surgeon's skill. In contrast to these reports, the strict control parameters were met in only 1 of the 21 patients in our study who underwent surgery; however, GH levels were reduced by 53.1% (12.7-83.0%) and IGF-1 levels were reduced by 69% (52.0-85.5%). Indeed, most of our patients had macroadenomas, and the cavernous sinus was involved in some cases. However, the surgical approach was important to preserve vision and reduce the tumor mass.

Conventional radiotherapy was performed in 10 patients and resulted in a considerable reduction in GH levels after surgery in 9 patients (Table 4). However, the treatment was not successful according to the current control parameters 6,7. The addition of SRLs at individualized doses, which has been shown in larger series to result in a success rate of 55.0% 15, 17, proved successful as complementary therapy.

Currently, 20 patients are undergoing follow-up (Table 6). Other clinical treatment options such as administration of cabergoline or lanreotide may increase the efficacy of treatment among patients who reach the maximum dose of octreotide or among noncompliant patients. Three patients complained of pain at the injection site and were found to be using the medication irregularly. Proper control was obtained when the treatment was switched to lanreotide as described by Strasburger et al. 21.

Another possible approach to treatment is the use of SRLs as an initial approach in macroadenoma cases to reduce the tumor size and allow better surgical outcomes 22-25. Currently, some patients are receiving this treatment. This approach can help the care provider decide on the appropriate time for surgery. Another point to consider is that the co-secretion of prolactin observed in 4 patients allowed them to obtain cabergoline from the SUS, which made the treatment more effective 2,4.

According to the literature, cardiovascular events are the main cause of death in acromegaly patients, indicating that cardiovascular risk should be monitored 26,27. In the present study, the percentage of subjects with hypertension, impaired fasting glucose, diabetes and dyslipidemia was similar to the percentage of such patients in the reports by Alexopoulou et al. 28 and Dreval et al. 29. However, in the 9 patients who died during the course of our study, infections were the main cause of unfavorable outcomes; this might have been related to inadequate control of aspects of acromegaly other than metabolic complications 26,27.

Based on the foregoing evaluation of our approach to the diagnosis and treatment of acromegaly, both screening for acromegaly and the treatment of chronic complications of acromegaly need to be more systematic and multidisciplinary. The importance of actively screening the general population for thyroid cancer, colon cancer, and breast cancer is supported by data from the literature 26,30,31 and has been well accepted by patients who are convinced of the importance of early diagnosis for healing.

Although quality of life tests were not applied in this study 7, we observed that one of the main factors that limited our patients' life-styles was joint and spinal injuries. These were present in most of our patients. The changes that result in such injuries are irreversible and result from increased GH and IGF-1 levels over long periods of undiagnosed illness 26. Greater involvement of colleagues in other specialties following diagnosis could help provide early and preventive treatment for various conditions involving the musculoskeletal system.

The present study has some limitations. These include the small size of the sample due to the rarity of the disease, the difficulty of comparing different treatment modalities that have changed over time and the need to analyze the data according to the current criteria, which demand stricter control of GH and IGF1 levels 6,7. On the other hand, this study has the strength to show that the resources currently available for the treatment of patients with acromegaly/gigantism allow them to be properly treated in regional reference centers.

We conclude that treatment of acromegaly represents a multi-professional challenge and that in our environment, it will be more effective and successful with earlier diagnosis, improvement in surgical techniques, regular follow-up and prompt availability of clinical treatment with SRL. In addition, multidisciplinary care of patients with chronic complications and the systematic search for data in pre-defined protocols 32 will improve the quality of life and reduce the cardiometabolic risk factors and mortality of patients with acromegaly.

## AUTHOR CONTRIBUTIONS

Borges MF and Lara BH developed the project, coordinated the work and wrote the manuscript. Ferreira BP, Borges MF and Lara BH guided the patients according to the methods reported in this work, performed the GH suppression tests and guided the careful treatment and follow-up of the patients. Bugiga FC, Soares JM and Tome JM collected the patients� records, plotted the data in tables and in Excel, performed the statistical analysis and configured the tables. Araujo LP, Dezena RA and Sousa JC performed the neurosurgeries and helped in writing the manuscript.

## Figures and Tables

**Table 1:** Clinical data of patients with acromegaly and gigantism.

Parameter	G1 (n=17)	G2 (n=13)	Total (n=30)
Age at diagnosis (yrs., mo.)	#45.5 (a) (14.0 – 67.0)	47.5 (a) (14.0 – 86.0)	46.0 (14.0 – 86.0)
Weight (kg)	87.3 (64.3 – 199.0)	71.2 (64.4 – 89.0)	-
Height (m)	1.77 (1.63 – 1.94)	1.59 (1.55 – 1.77)	-
BMI (kg/m^2^)	28.4 (b) (17.6 – 53.4)	27.8 (b) (25.5 – 29.8)	29.0 (17.6 – 53.4)
Follow-up period (yrs.)	10.0 (1.0 – 20.0)	7.0 (0.4 – 23.0)	9.0 (0.4 – 23.0)

#Data are expressed as median (minimum and maximal values)

Student's t-test

a: *p*=0.973

b: *p*=0.425.

**Table 2:** Clinical symptoms and signs obtained from medical records and frequently reported by acromegaly patients at diagnosis.

Clinical feature	G1 (n=17)	G2 (n=13)	Total	
			(n=30)	%
Acral overgrowth	15	12	27	90.0
Bone and muscle pain	11	6	17	56.7
Headaches	11	5	16	53.3
Visual field deficits	5	6	11	36.7
Weight gain	4	5	9	30.0
Nasal obstruction/sinusites	6	3	9	30.0
Edema/skin thickening	4	4	8	26.7
Constipation	3	4	7	23.3
Adynamia	2	5	7	23.3
Voice deepening	4	2	6	20.0
Amenorrhea / Galactorrhea	–	6	6	20.0
Impotence	4	–	4	13.3
Tall stature	2	1	3	10.0

**Table 3:** Therapeutic approaches used in 28 patients with acromegaly and gigantism.

	G1 (n=16)	G2 (n=12)	Total	
			(n=28)	%
Surgery^b^+SRLs^c^	7	4	11	39.3
Surgery+radiotherapy+SRLs	4	1	5	17.8
Surgery+radiotherapy	1	2	3	10.7
SRL+cabergoline	1	2	3	10.7
SRL	2	1	3	10.7
Surgery+radiotherapy+SRLs+cabergoline	0	1	1	3.6
Surgery	0	1	1	3.6
Radiotherapy	1	0	1	3.6

a: two of 30 patients were not treated, 1 died and another refused treatment after diagnosis.

b: transphenoidal surgery

c: SRLs= somatostatin receptor ligands.

**Table 4:** Decrease in serum growth hormone concentrations [GH] from pretreatment values in patients who received different modalities of treatment.

Treatment performed	↓ [GH] ng/mL	↓ [GH] %
Pretreatment GH^(a)^ (n=21)	25.6 (8.0 – 355.0)	–
After surgery ^(b)^ (n=21)	14.5 (1.5 – 310.0)	53.1 (12.7 – 83.0)
Surgery + RT ^(c)^ (n=9)	8.10 (1.6 – 79.2)	87.7 (14.0 – 96.8)
Surgery + RT + SRLs ^(d)^ (n=6)	0.7 (0.3 – 8.7)	98.7 (90.4 – 99.5)
Surgery + SRLs ^(e)^ (n=11)	0.6 (0.2 – 7.9)	95.2 (65.0 – 99.9)

RT= radiotherapy; SRLs= somatostatin receptor ligands

Friedman's test followed by Dunn's test

a x b = *p*>0.05 (n=21)

a x b x c = *p*>0.05 (n=9)

a x b x c x d = *p*< 0.03; d < a, b, c (n=6); % values = d > a, b, c

Friedman's test followed by Dunn's test

a x b x e = *p*<0.05; e < b < a (n=11); % values = e > b

Mann-Whitney test

d x e = *p*>0.05; % values = *p* >0.05

c x e = *p* <0.05; e < c; % values = e > c.

**Table 5:** Therapeutic approach used in 20 patients in regular follow-up.

	G1	G2	Total	
			N	%
Surgery^a^+SRLs^b^	7	2	9	45.0
Surgery+ratiotherapy+SRLs	2	1	3	15.0
SRLs+cabergoline	1	2	3	15.0
Surgery+radiotherapy+SRLs+cabergoline	0	1	1	5.0
SRLs	2	0	2	10.0
Surgery	0	1	1	5.0
Surgery+radiotherapy	0	1	1	5.0

a: transphenoidal surgery

b: SRLs= somatostatin receptor ligands.

**Table 6:** Growth hormone (GH) and insulin-like growth factor 1 (IGF-1) concentrations in 20 treated patients at regular follow-up.

GH (ng/mL)	IGF-1 (ng/mL)	G1 (n)	G2 (n)	Total	
				N	%
<1	nl	8	3	11	55.0
<1	↑	1	1	2	10.0
>1 – <2.5	↑	0	2	2	10.0
>2.5	↑	4	1	5	25.0
